# Genome-wide identification of the potato WRKY transcription factor family

**DOI:** 10.1371/journal.pone.0181573

**Published:** 2017-07-20

**Authors:** Chao Zhang, Dongdong Wang, Chenghui Yang, Nana Kong, Zheng Shi, Peng Zhao, Yunyou Nan, Tengkun Nie, Ruoqiu Wang, Haoli Ma, Qin Chen

**Affiliations:** Department of Agronomy, State Key Laboratory of Crop Stress Biology for Arid Areas, Northwest A & F University, Yangling, Shaanxi, China; Hainan University, CHINA

## Abstract

WRKY transcription factors play pivotal roles in regulation of stress responses. This study identified 79 WRKY genes in potato (*Solanum tuberosum*). Based on multiple sequence alignment and phylogenetic relationships, WRKY genes were classified into three major groups. The majority of WRKY genes belonged to Group II (52 *StWRKYs*), Group III had 14 and Group I consisted of 13. The phylogenetic tree further classified Group II into five sub-groups. All *StWRKY* genes except *StWRKY79* were mapped on potato chromosomes, with eight tandem duplication gene pairs and seven segmental duplication gene pairs found from *StWRKY* family genes. The expression analysis of 22 *StWRKY*s showed their differential expression levels under various stress conditions. *Cis*-element prediction showed that a large number of elements related to drought, heat and salicylic acid were present in the promotor regions of *StWRKY* genes. The expression analysis indicated that seven *StWRKY*s seemed to respond to stress (heat, drought and salinity) and salicylic acid treatment. These genes are candidates for abiotic stress signaling for further research.

## Introduction

Transcription factors (TFs) participate in gene transcription regulatory networks that regulate gene expression in plants. In the plant genome, a large number of genes encode TFs[1].

The WRKY TF family, named because of containing the WRKY domain, exists widely in many organisms [2]. The WRKY domain is a highly conserved sequence of 60 amino acids [3], heptapeptide WRKYGQK which also has WRKYGKK, WKKYGQK, WRKYGQR, WRKYGEK and some other forms [4, 5], and a zinc finger structure. which is itself divided into two types, C2H2 and C2HC [6]. According to the number of WRKY domains and the type of zinc finger, the WRKY family is divided into three main groups [7]. Group I contains two WRKY domains located in the C- and N-terminus, respectively. The other two groups have just one WRKY domain. Groups I and II have the C2H2-type zinc finger, and only Group III has the C2HC-type. In *Arabidopsis thaliana*, Group II genes are also divided into five sub-groups: II-a, II-b, II-c, II-d and II [8].

The first WRKY TF, which was reported to be a DNA binding protein, *SPF1*, was cloned from sweet potato [9] and WRKY TF families have since identified [10]. Previous studies identified at least 72 WRKY family genes in *Arabidopsis thaliana* [8], about 100 in *Populus* [11], 59 in *Vitis vinifera* [12], 81 in *Solanum lycopersicum* [13] and 71 in *Capsicum annuum* [14]. Previous studies of WRKY family evolution showed that Group I was an ancestral group. The descent of Group II and III were origin from group I which were lack of an N-terminal WRKY domain [15].

Many studies have shown that WRKY gene family members are related to plant development processes, such as fiber development [16] and leaf senescence in cotton [17]. Some researchers showed that the WRKY gene family members were related to development of the embryo and anther [18, 19]. They were also played roles in plant stresses. WRKY genes can defend against infections by bacteria [20], fungi [11] and viruses [20] and provide resistance to cold [12], salt [21], drought [22] and wounding [23]. The research indicated that 11 *OsWRKY* genes were responses to salt, drought, cold and heat stresses by Qin [24]. Similarly, several *GmWRKY* genes through the transgenic *Arabidopsis* plants to improve the resistance in these stresses [25]. Identification of WRKY genes function in stresses could increase stress resistance to breed new cultivars.

There has been a little relevant research on the WRKY family in the potato. As one of the popular foods in the world, especially in Europe and America, many potatoes are consumed each year. To satisfy this demand, enhancing yield is increasingly important. Studies of planting microenvironment have shown that abiotic and biotic stresses are important factors restraining potato yield [26]. However, *StWRKY*1 has only been researched by Yogendra [27] and Shahzad [28]. And some WRKY genes related to arbuscular mycorrhizal potato root colonization have been reported [29]. In 2016, Yogendra showed the *StWRKY8* was related to blight [30].As a stress-related gene family, the possibility of using them to improve the stress resistance and adaptation should be researched. This study aimed to survey the information about WRKY genes in *S*. *tuberosum* and the expression for several StWRKY genes under stress conditions.

## Materials and methods

### Database sequence searches

The potato protein and gene sequences were downloaded from the PGSC database (http://solanaceae.plantbiology.msu.edu/pgsc_download.shtml). The *Arabidopsis* WRKY and *S*. *lycopersicum* WRKY locus numbers were acquired from papers by Eulgem [8] and Huang [13], respectively. The WRKY protein sequences were obtained from the TAIR (https://www.arabidopsis.org/tools/bulk/sequences/index.jsp) and plantTFDB (http://planttfdb.cbi.pku.edu.cn/) websites.

*AtWRKY* and *SlWRKY* protein sequences were used as queries to search the *Solanum tuberosum* WRKY family members using BLASTP [31]. To achieve accurate results, the E value was set at 10. Then the website NCBI-CDD (http://www.ncbi.nlm.nih.gov/cdd) was used to analyze the conserved sequences and to remove sequences that lacked WRKY annotation. The software cluster X [32, 33] was used to ensure existence of the WRKYGQK conserved domain or similar sequences.

### Mapping potato WRKY genes on chromosomes

Relevant information on all identified *StWRKYs* was found on the PGSC website (http://solanaceae.plantbiology.msu.edu/index.shtml). The software MapChart2.30 was used to map the gene locus on chromosomes. Tandem duplication of *StWRKY*s was identified according the method of Cheng [3].Two or more WRKY genes within a distancs of 100 kb were regarded as tandem duplications [3, 34]. Information on segmental duplications was sourced from the PGDD website (http://chibba.agtec.uga.edu/duplication/), and segmental duplications were displayed using Circos software.

### Constructing the phylogenetic tree and classification of WRKY members

To build the *StWRKY* phylogenetic tree and classify them into groups required information on some other plants’ WRKY proteins. The *Arabidopsis*, *C*. *annuum* and *S*. *lycopersicum* WRKY proteins were acquired from other research [8, 13, 35]. All of these protein sequences were assessed with Clustal X software and complete alignment performed. Then, the phylogenetic tree was constructed by Test Neighbor-Joining Tree Method [36] using MEGA6.0 software. DNAMAN software was used to analyze sequence alignments and identify *StWRKY*.

### Structures of WRKY members

The exon-intron structures of potato WRKY genes were determined using the GSDS2.0 website (http://gsds.cbi.pku.edu.cn/index.php) [37]. The motifs were analyzed using the MEME website (http://meme-suite.org/index.html) [4]. In total, 20 motifs were searched per member.

### Plant growth conditions and treatments

The potato material was a sequenced double-haploid variety, DM1-3-516-R44. Virus-free seedlings were grown in a plant incubator at 25±1°C under a 10 000 Lx in light/dark for 16/8 h with solid Murashige & Skoog Basal Medium with Vitamins (MS, USA) culture medium for 4 weeks. Then the seedling transferred into MS liquid medium.

Potential heat-, drought- and salt- related WRKY genes in potato were identified in potato. 150 mM NaCl was added for salt treatment, and 260 mM Mannitol was added for drought-treatment. The seedlings were maintained at 35°C for heat-treatment. For the biotic stress, salicaylic said (SA) was sprayed on potato leaf surfaces after growing in greenhouse for 4 weeks. Bring the treatment-plants into culture dish with SA liquid (10 μM in water) filter paper. All processing materials were collected at 3 and 24 h.

### Quantitative real-time PCR (qRT-PCR) analysis

After 3 h and 24 h plant shoot were harvested and total RNA was isolated from the various treated samples using the Plant RNA Purification Reagent (Invitrogen, USA) And cDNA was synthesized using thePrimeScript reagent kit (Takara, Japan). In general, the final concentration of cDNA was diluted five-fold for use. Primer Premier 5 software designed the gene-specific primers for qRT-PCR reactions. Bio-Rad Real-Quantitative real-time PCR analysis Time System (CFX96, USA) was used to analysis which was based on three technical replicates. The expression levels of gene from the diverse treatment were normalized using a reference gene, *elongation factor 1-a (ef1a)*[38]. The relative expression levels were analyzed from the method of Haoli Ma [39].

### *Cis*-element analysis of *StWRKY* genes

The gene upstream sequences (approximately 1500bp) of 22 *StWRKY*s were searched in the Phytozome 12 website (https://phytozome.jgi.doe.gov/pz/portal.html), and *cis*-elements were acquired from the plantCARE website (http://bioinformatics.psb.ugent.be/webtools/plantcare/html/). The mainly focused in ABRE (*cis*-acting element involved in the abscisic acid responsiveness), HSE (*cis*-acting element involved in heat stress responsiveness), MBS (MYB binding site involved in drought-inducibility), TC-rich repeats (*cis*-acting element involved in defense and stress responsiveness), TCA-element (*cis*-acting element involved in salicylic acid responsiveness) and W-box (WRKY binding site) [35].

## Results

### Identification of *StWRKY* family genes

To identify the WRKY TF genes in potato, the nonredundant proteins were used to identify the WRKY family members through BLASTP search. A total of 761 unique sequences had high similarity with *Arabidopsis* WRKY genes and 147 unique sequences had high similarity with tomato WRKY genes. Following the analysis for NCBI-CDD website, 129 sequences were acquired as the putative WRKY members. However, this result contained the non-representative sequences. Furthermore, WRKY family members should contain a conserved heptapeptide [40], but sequences alignment showed that some sequences did not have a WRKYGQK or WRKYGQK-like conserved domain [4, 40]. Following ClustalX analysis of full-length protein sequences, 79 unique sequences were acquired and relevant information obtained (Table 1). Although the WRKYGQK motif was a highly conserved domain, but 12 genes were also existed a part of variations in amino acids (Table 1).

**Table 1:** Identification of WRKY genes in potato.

NAME	Gene locus	chromosome Location	WRKY domain			Group	PI	Mw (KD)	Protein length(aa)	CDS length(bp)	Extron
			Conserved heptapeptide	Zinc-finger type	Domain number						
StWRKY01	PGSC0003DMG400011457	chr01	WRKYGQK	C2H2	1	IIc	4.97	37.28	339	1020	3
StWRKY02	PGSC0003DMG400009014	chr01	WRKYGQK	C2H2	1	IIe	5.2	27.22	244	735	3
StWRKY03	PGSC0003DMG400009051	chr01	WRKYGQK	C2H2	1	IIc	6.07	35.88	317	954	3
StWRKY04	PGSC0003DMG400031175	chr01	WRKYGQK	C2H2	1	IIc	8.16	27.50	251	756	4
StWRKY05	PGSC0003DMG400000064	chr01	WRKYGQK	C2H2	1	IIe	5.73	38.83	352	1059	3
StWRKY06	PGSC0003DMG400000211	chr01	WRKYGQK	C2HC	1	III	5.64	38.87	346	1041	3
StWRKY07	PGSC0003DMG400029779	chr01	WRKYGQK	C2H2	1	IIb	8.14	45.94	413	1242	4
StWRKY08	PGSC0003DMG400015104	chr02	WRKYGQK	C2H2	1	IIe	5.16	46.16	413	1242	3
StWRKY09	PGSC0003DMG400022063	chr02	WRKYGQK	C2H2	1	IIb	7.02	55.21	496	1491	5
StWRKY10	PGSC0003DMG400025481	chr02	WRKYGQK	C2H2	1	IId	9.12	37.38	334	1005	3
StWRKY11	PGSC0003DMG402006935	chr02	WRKYGQK	C2H2	1	IIb	6.29	48.68	443	1332	3
StWRKY12	PGSC0003DMG400009703	chr02	WRKYGQK	C2H2	1	IIc	6.49	36.17	318	957	3
StWRKY13	PGSC0003DMG400028469	chr02	WRKYGQK	C2H2	1	IIe	5.94	33.57	299	900	3
StWRKY14	PGSC0003DMG400016441	chr02	WRKYGQK	C2H2	1	IIb	6.98	60.06	553	1662	6
StWRKY15	PGSC0003DMG400024961	chr02	WRKYGQK	C2H2	1	IId	9.70	36.01	324	975	3
StWRKY16	PGSC0003DMG400001434	chr02	WRKYGQK/WRKYGQK	C2H2	2	I	6.13	50.16	451	1356	4
StWRKY17	PGSC0003DMG400020206	chr02	WRKYGQK	C2H2	1	IIc	9.52	16.45	141	426	2
StWRKY18	PGSC0003DMG401005575	chr03	WRKYGQK/WRKYGQK	C2H2	2	I	6.56	57.08	516	1551	4
StWRKY19	PGSC0003DMG400020608	chr03	WRKYGQK	C2HC	1	III	6.17	31.89	277	834	3
StWRKY20	PGSC0003DMG400041197	chr03	WRKYGQK	C2H2	1	IIe	8.46	28.69	249	750	3
StWRKY21	PGSC0003DMG400039175	chr03	WRKCGQK*	C2H2	1	IIe	8.89	28.66	249	750	3
StWRKY22	PGSC0003DMG400009103	chr03	WRKYGQK	C2HC	1	III	5.90	40.31	356	1071	3
StWRKY23	PGSC0003DMG400044842	chr03	WRKYGMK*	C2H2	1	IIe	6.06	29.51	259	780	1
StWRKY24	PGSC0003DMG400034476	chr03	WKKHGSN*	C2H2	1	IIe	7.06	19.99	173	552	1
StWRKY25	PGSC0003DMG400018081	chr03	WRKYGQK	C2H2	1	IIb	6.82	58.45	525	1578	5
StWRKY26	PGSC0003DMG400019824	chr03	WRKYGQK	C2H2	1	IIa	8.74	39.76	355	1068	5
StWRKY27	PGSC0003DMG400040494	chr04	WRKYGQK	C2H2	1	IIe	8.86	35.67	311	936	5
StWRKY28	PGSC0003DMG401031196	chr04	WRKYGKK*	C2H2	1	IIc	9.47	16.12	138	417	3
StWRKY29	PGSC0003DMG400019706	chr04	WRKYGQK	C2H2	1	IIc	9.00	27.26	234	705	3
StWRKY30	PGSC0003DMG400031140	chr04	WRKYGKK*	C2H2	1	IIc	6.55	26.68	231	696	3
StWRKY31	PGSC0003DMG400007947	chr04	WRKYGQK	C2H2	1	IId	9.67	38.88	354	1065	3
StWRKY32	PGSC0003DMG400028335	chr05	WRKYGQK/WRKYGQK	C2H2	2	I	8.05	55.46	508	1527	4
StWRKY33	PGSC0003DMG400028381	chr05	WRKYGQK	C2H2	1	IIc	5.34	35.28	326	981	3
StWRKY34	PGSC0003DMG400021895	chr05	WRKYGQK	C2H2	1	IIc	9.22	19.92	172	519	2
StWRKY35	PGSC0003DMG400036639	chr05	WRKYGQK	C2H2	1	IIe	9.53	37.44	330	993	3
StWRKY36	PGSC0003DMG400035855	chr05	WRKYGQK	C2H2	1	IIe	9.04	37.55	330	993	3
StWRKY37	PGSC0003DMG400033884	chr05	WRKYGQR*	C2HC	1	III	6.25	32.92	294	885	3
StWRKY38	PGSC0003DMG401033880	chr05	WRKYGQK	C2HC	1	III	5.83	37.37	331	996	3
StWRKY39	PGSC0003DMG400017990	chr05	WRKYGQK	C2HC	1	III	5.48	34.06	301	906	3
StWRKY40	PGSC0003DMG400027208	chr05	WRKYGQK	C2H2	1	IIc	9.01	28.14	250	753	3
StWRKY41	PGSC0003DMG400023360	chr05	WRKYGQK/WRKYGQK	C2H2	2	I	6.84	51.90	457	1374	3
StWRKY42	PGSC0003DMG400005329	chr06	WRKYGQK	C2H2	1	IId	9.62	39.95	355	1068	3
StWRKY43	PGSC0003DMG400008776	chr06	WRKYGQK	C2HC	1	III	5.94	38.15	347	1044	4
StWRKY44	PGSC0003DMG400027582	chr06	WRKYGQK	C2HC	1	III	8.06	26.02	223	672	3
StWRKY45	PGSC0003DMG400016769	chr06	WRKYGQK/WRKYGQK	C2H2	2	I	6.90	59.57	534	1605	5
StWRKY46	PGSC0003DMG400028520	chr06	WRKYGQK	C2H2	1	IIa	8.37	39.79	360	1083	4
StWRKY47	PGSC0003DMG400006155	chr07	WRKYGQK/WRKYGQK	C2H2	2	I	6.55	46.54	422	1269	4
StWRKY48	PGSC0003DMG400015015	chr07	WRKYGQK	C2H2	1	IIb	6.09	60.26	557	1674	5
StWRKY49	PGSC0003DMG400020432	chr07	WRKYGQK	C2H2	1	IIe	5.51	28.19	256	771	4
StWRKY50	PGSC0003DMG400017349	chr07	WRKYGQK	C2H2	1	IIc	6.26	36.29	319	960	3
StWRKY51	PGSC0003DMG400022290	chr07	WRKYGQK/WRKYGQK	C2H2	2	I	7.05	64.91	596	1791	6
StWRKY52	PGSC0003DMG400022143	chr07	WRKYGQK/WRKYGQK	C2H2	2	I	6.01	81.04	748	2247	5
StWRKY53	PGSC0003DMG400009530	chr08	WRKYGQK	C2H2	1	IId	9.62	36.33	334	1005	3
StWRKY54	PGSC0003DMG400005835	chr08	WRKYGQK	C2HC	1	III	5.69	40.74	360	1083	3
StWRKY55	PGSC0003DMG400005836	chr08	WRKYGQK	C2HC	1	III	6.89	34.07	305	918	3
StWRKY56	PGSC0003DMG400008188	chr08	WRKYGKK*	C2H2	1	IIc	4.79	14.65	128	387	2
StWRKY57	PGSC0003DMG402007388	chr08	WRKYGQK	C2H2	1	IIa	8.84	28.72	253	762	4
StWRKY58	PGSC0003DMG400007387	chr08	WRKYGQK	C2H2	1	IIa	5.70	29.74	261	786	4
StWRKY59	PGSC0003DMG400012318	chr08	WRKYGQK	C2H2	1	IIe	5.57	35.04	307	924	3
StWRKY60	PGSC0003DMG400012317	chr08	WRKYGQK	C2H2	1	IIc	9.13	26.17	228	687	2
StWRKY61	PGSC0003DMG400012160	chr08	WRKYGQK	C2HC	1	III	5.40	38.57	339	1020	3
StWRKY62	PGSC0003DMG400011633	chr09	WRKYGQK/WRKYGQK	C2H2	2	I	7.05	45.47	408	1227	4
StWRKY63	PGSC0003DMG400029207	chr09	WRKYGQK	C2HC	1	III	5.63	33.46	293	882	3
StWRKY64	PGSC0003DMG400015076	chr09	WRKYGQK	C2H2	1	IId	9.65	39.10	346	1041	3
StWRKY65	PGSC0003DMG400011271	chr10	WIKYGEN/WRKYGQK	C2H2	2	I	5.07	75.77	697	2094	5
StWRKY66	PGSC0003DMG401010558	chr10	WRKYGQK	C2H2	1	IIe	8.36	23.24	208	627	3
StWRKY67	PGSC0003DMG400019884	chr10	WRKYGQK	C2HC	1	III	5.55	33.52	290	873	3
StWRKY68	PGSC0003DMG400019408	chr10	WRKYGQK	C2H2	1	IIe	4.73	26.70	238	717	3
StWRKY69	PGSC0003DMG400008391	chr10	WRKYGQK	C2HC	1	III	8.94	25.43	218	657	3
StWRKY70	PGSC0003DMG400010987	chr10	WRKYGQK/WRKYGQK	C2H2	2	I	9.51	52.05	467	1404	5
StWRKY71	PGSC0003DMG400007788	chr12	WRKYGKK*	C2H2	1	IIc	6.56	37.68	334	1005	3
StWRKY72	PGSC0003DMG400037700	chr12	WHKCGQK*	C2H2	1	IIe	8.11	37.51	331	996	3
StWRKY73	PGSC0003DMG402028822	chr12	WRKYGQK/WRKYGQK	C2H2	2	I	6.62	60.48	549	1650	4
StWRKY74	PGSC0003DMG400029815	chr12	WRKYGQK/WRKYGQK	C2H2	2	I	6.37	65.80	611	1836	6
StWRKY75	PGSC0003DMG400028633	chr12	WRKYGQK	C2H2	1	IIa	9.52	24.70	217	654	4
StWRKY76	PGSC0003DMG400023196	chr12	WRKYGKK*	C2H2	1	IIc	8.28	24.90	213	642	3
StWRKY77	PGSC0003DMG400016957	chr12	WRKYGKK*	C2H2	1	IIc	9.35	14.57	124	375	2
StWRKY78	PGSC0003DMG400029371	chr12	WRKYGQK	C2H2	1	IId	9.61	34.03	312	939	3
StWRKY79	PGSC0003DMG400033177	chr00	WRKYGKK*	C2H2	1	IIc	9.23	12.10	102	309	2

Notes:

* WRKY conserved sequence is WRKYGQK-like that is indicated by asterisk.

Chr00 means this sequence has not located.

The average *StWRKY* proteins length was 338 aa and in the range of 102 aa (*StWRKY79*) to 748 aa (*StWRKY52*). Information on gene loci number, chromosome location, WRKYGQK conserved heptapeptide, zinc-finger motif type, number of WRKY domains, isolelectrical point (PI), molecular weight (Mw) and number of exons are given in Table 1.

### Chromosomal location of *StWRKY* members and analyze the gene duplication

**Fig 1:**
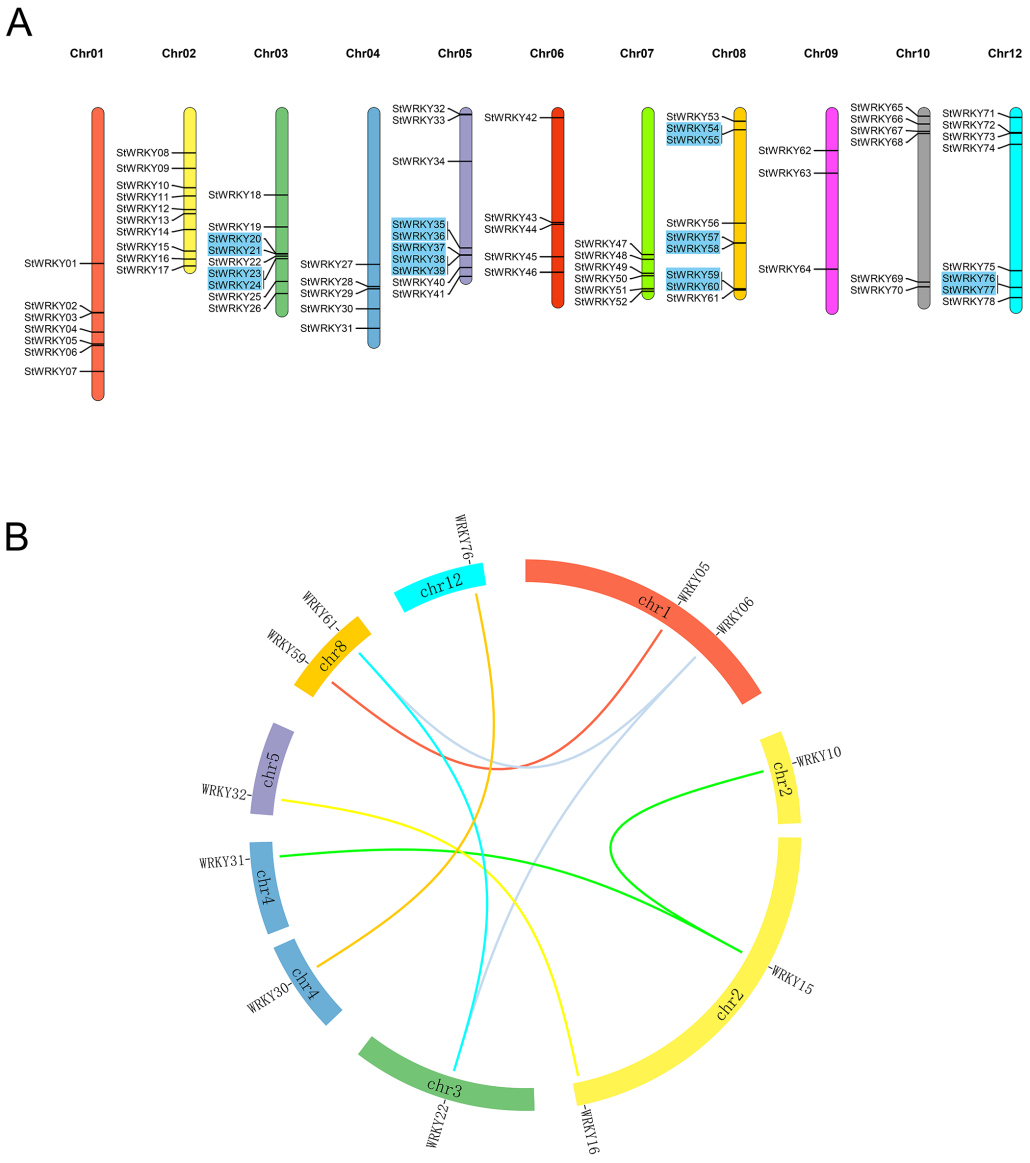
Located the *StWRKY* members in potato chromosomes and analyzed their duplications. (A) The location of *StWRKY* members on potato chromosomes and the tandem duplication. The blue boxes indicate these members are tandem duplications. And *StWRKY79* is not located in any chromosomes. (B) The segmental duplication about *StWRKY* members. The different color blocks indicate the part of potato chromosomes. The thick lines with the same colors about chromosomes mean the tandem duplications and the fine lines are connected the segmental duplication pairs and different colors means different duplication.

Mapchart software was used to determine the location of the gene on each chromosome (Fig 1A). According to the position of genes in chromosomes, we were named these WRKY genes from 1 to 79. The map showed that chromosomes contained all *StWRKY*s, except *StWRKY79*. Two shorter chromosomes, the chromosome 2 (48.61 Mb) and the chromosome 5 (52.07 Mb) had 10 genes, respectively. Only 3 genes were occurred in the chromosome 9. And none located in the chromosome 11. The event of genes located closely in some chromosomes.

Through identification of tandem duplication was based on Cheng’s method and these closely genes were termed a cluster [3]. *StWRKY* genes formed in clusters on chromosome 3, 5, 8 and 12. A total of 8 clusters (17 genes) confirmed in blue boxes (Fig 1A). The research of the database which downloaded from PGSC website showed seven parts from *StWRKY* genes with segmental duplications (15 genes) (Fig 1B) and these were distributed on seven chromosomes.

### Phylogenetic analysis and classification of *StWRKY* genes

**Fig 2:**
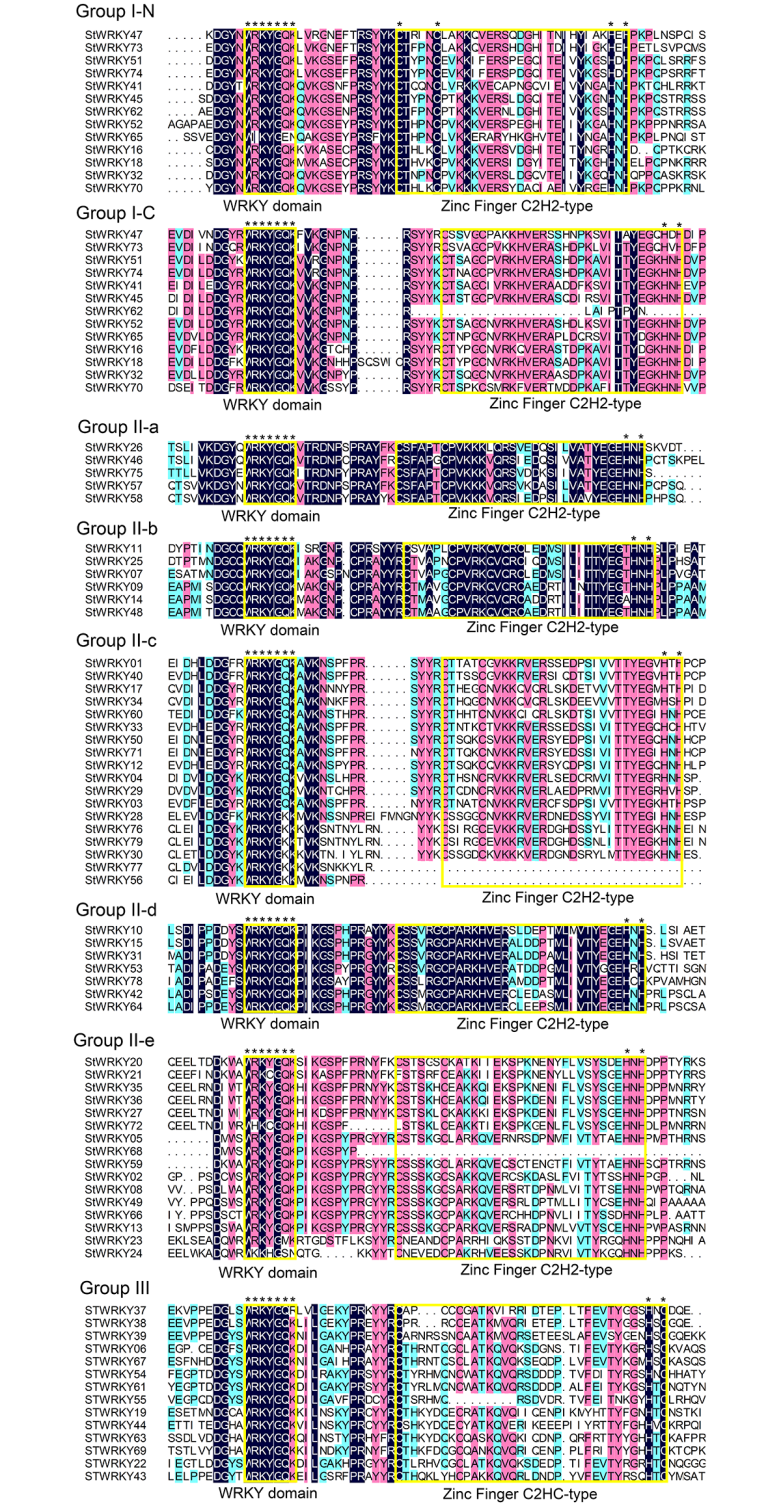
Comparison of the protein sequences from 79 *StWRKY* proteins. Alignment was using software DNAMAN. The N or C indicates the N-terminal and the C-terminal, respectively. The amino acids forming the zinc-finger motif and WRKY domain are covered in yellow box. The asterisk indicated the main conversed sequences.

The defining characteristics of the WRKY family are the WRKYGQK domain and zinc-finger structure. However, different group owned their specific components. The detail structures about WRKY domain and zinc finger type could be displayed by multiple sequence alignment (Fig 2). A total of 13 genes contained two WRKYGQK domains were divided into one group, whereas the zinc finger had slight differences, one was CX4CX22HXH and another was CX4CX23HXH. The heptapeptide WRKYGQK co-located in C- and N-terminal, respectively. Thus, Group I also could be separated into I-C and I-N [41], even though they were be in same gene. The other part was constructed by 14 genes which could be easily differentiated by presence of special zinc finger, CX7CX24HXC [42]. The remaining genes (52 genes) formed the last part, a large genes containing one WRKY domain and an adjacent CX5CX23HXH zinc finger structure [8]. Interestingly, four *StWRKY*s which did not contain a whole zinc finger motif structure also belonged to the WRKY family.

**Fig 3:**
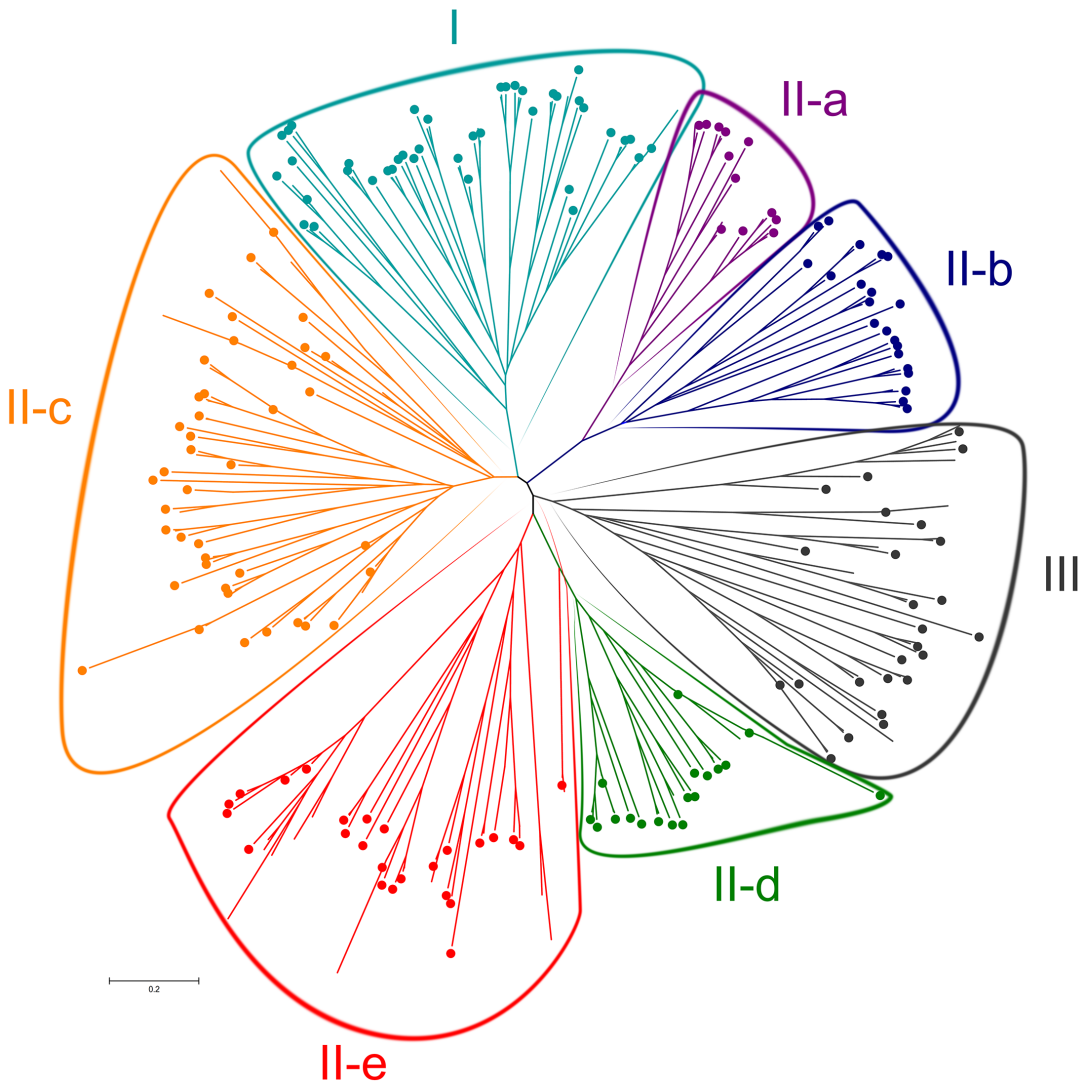
Phylogenetic tree builds for all WRKY proteins from 4 plants. *Arabidopsis*, *C*. *annuum*, *S*. *lycopersicum* and *S*. *tuberosum*, totally 261 protein sequences build the tress using the Neighbor-Joining method. Different regions distinguish the groups.

In order to acquire a detailed classification, four kinds of plant WRKY members were used to construct the phylogenetic tree: 54 WRKY proteins from *A*. *thaliana*, 53 WRKY proteins from *C*. *annuum*, 77 from *S*. *lycopersicum* and 79 from *S*. *tuberosum* (Fig 3). Following others categories, Group II was classified into five sub-groups: Group II-a (5 members), II-b (6), II-c (18), II-d (7), and II-e (16). Group II-a and II-b, and Group II-d and II-e, were clustered in a branch, respectively. The classification was the same as found in other higher plants, especially in dicotyledons [8].

### Structures of *StWRKY* gene family

**Fig 4:**
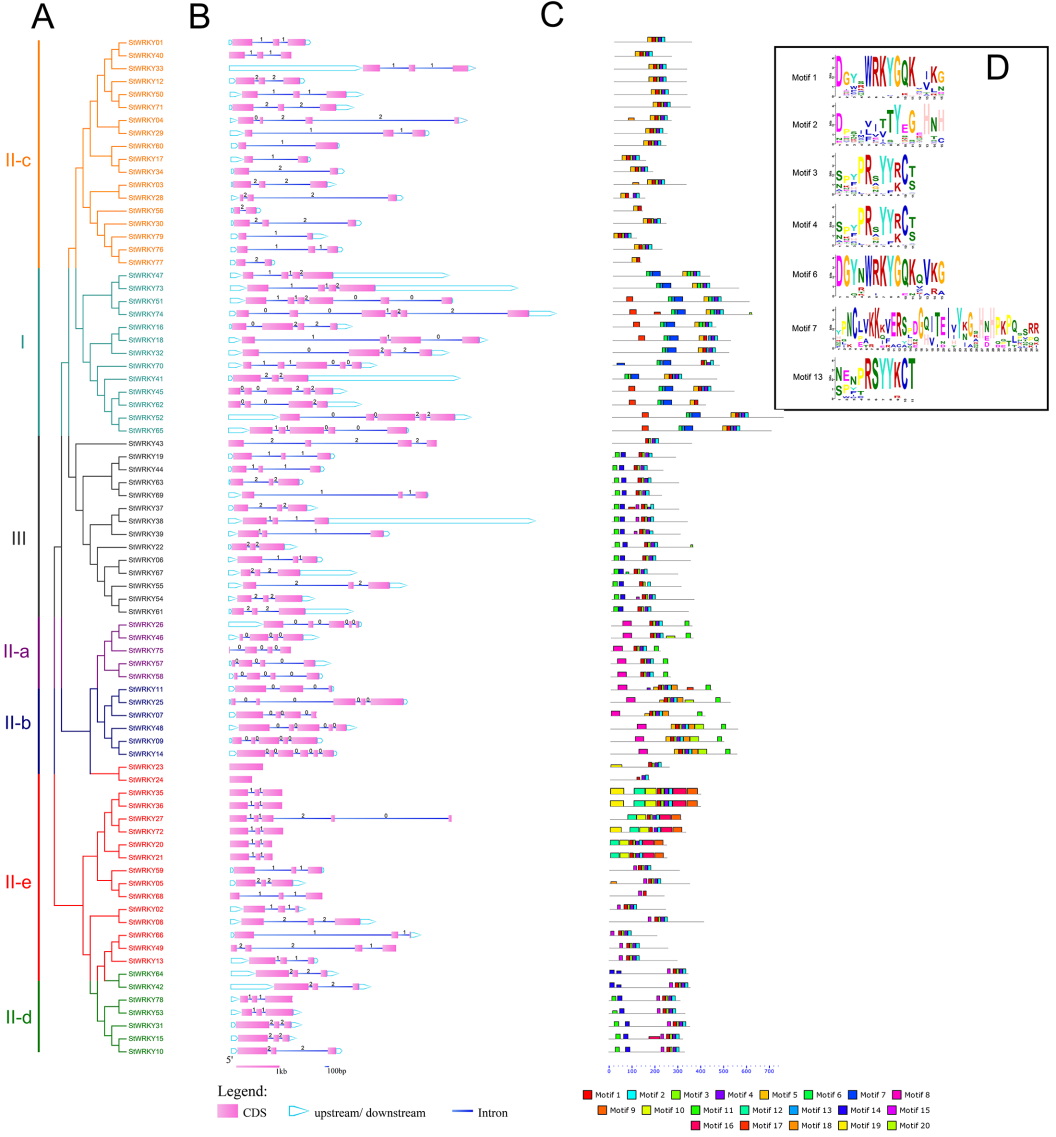
Structure analyzes *StWRKY* members. The WRKY members are divided into 7 groups. (A) Phylogenetic tree of *StWRKY* members. Neighbor-Joining tree constructed with WRKY proteins in potato. (B) Intron-exon structure of *StWRKY genes*. The purple blocks indicate CDS, the black lines indicate introns and the blue blocks indicated upstream or downstream. (C) Distribution of conserved motifs of *StWRKY*s. (D) The sequences of 7 main motifs.

To better understand the similarity and diversity of gene motifs in different genes, 20 motifs were examined within genes using the MEME website (Fig 4C and 4D). Motif 1 and 6 were WRKYGQK or WRKYGQK-like domains and were broadly distributed in every *StWRKY* gene. Motif 6, 7 and 13 together were formed the main structure of N-terminal WRKY domain, and motif 1–4 comprised the C-terminal WRKY domain. Almost every gene contained motif 1–4. In addition, 13 genes had both motifs 1 and 6 [35]. The WRKY family was divided into 3 parts following the motifs species and combinations. The members in first part had motif 6, 7 and 13 and these members comprised a group. In the other group, there were 14 *StWRKY* genes which had variation in motif 2 compared with other members, the last amino acid residue H was instead C. This transformation led to the change of zinc finger type from C2H2 to CH2C. The 52 members belonged to a group was different with others (Fig 4A and 4C). The division of these members was same as phylogenetic tree.

The concept of intron–exon was first introduced due to the discovery that transcription sequences were usually longer than gene sequences [41]. Analysis using the GSDS2.0 website showed that almost every gene contained at least one intron in *StWRKY* genes and most genes usually had 1–5 introns. However, two members (*StWRKY23* and *StWRKY24*) had no introns. About one-half of *StWRKY*s possessed two introns. Furthermore, six members just had one intron, and 26 had more than three. The analysis showed that introns of *StWRKY* members had three phases (Fig 4B): 0, 1 and 2. Phases 1 and 2 contained more than a half of the WRKY genes. 18 genes had phase 0 only. Just *StWRKY38* gene had downstream sequence and *StWRKY52* gene had upstream sequence through the analysis.

### Expression analysis of *StWRKY* genes in response to stresses

**Fig 5:**
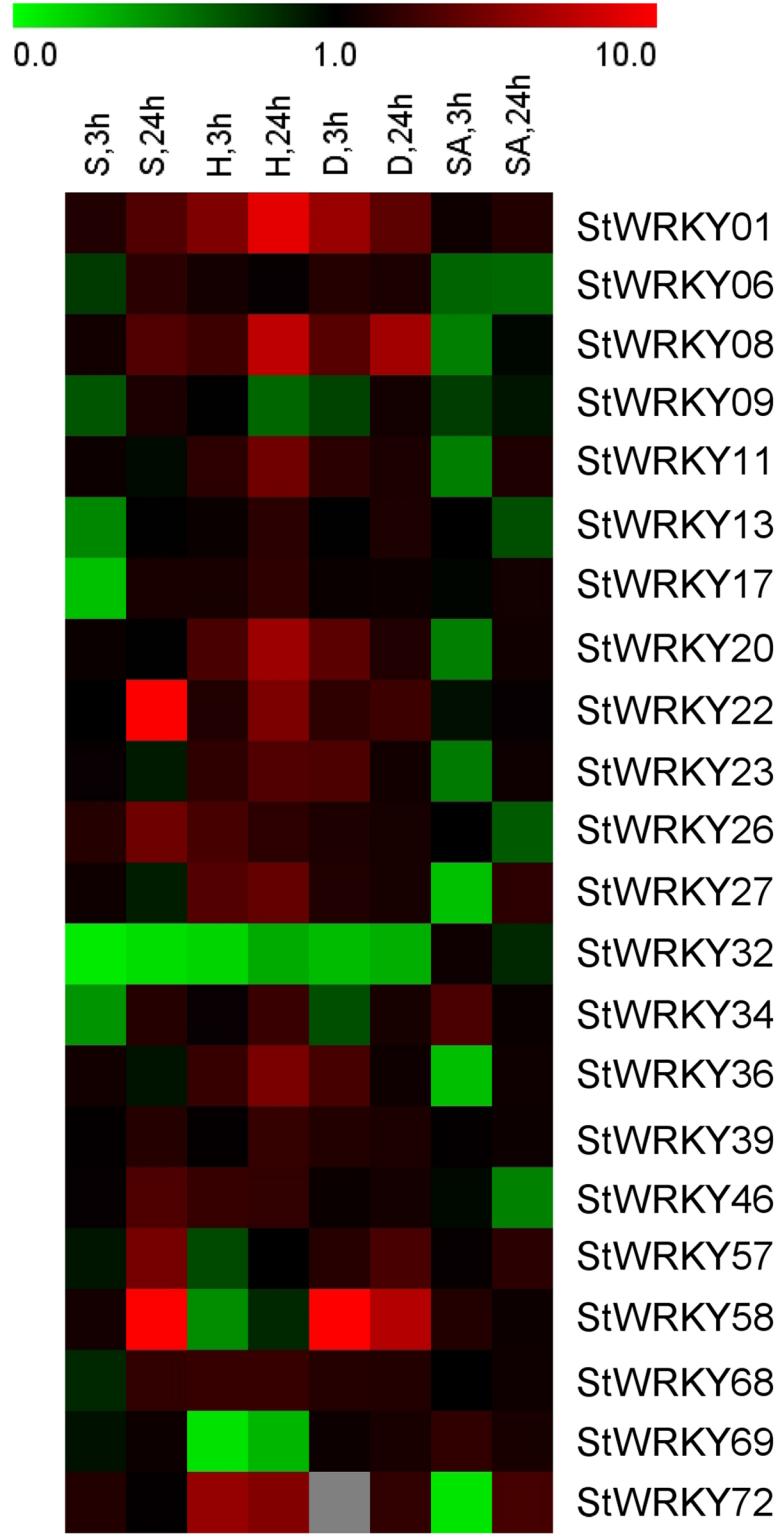
The expression pattern of 22 *StWRKY*s in four different stresses. S3 and S24 mean the salt treatment at 3 h and 24 h. H is Heat shorthand, D indicates Drought, and SA means salicylic acid. The data lower than 1.0 represents the down-regulated and higher 1.0 is up-regulated. The grey box indicates the *StWRKY72* cannot show detectable expression under drought-treatment in 3 h.

To demonstrate the function of WRKY genes and obtain information for future research, qRT-PCR analysis was used to reveal the expression of WRKY genes under stress. Using information from RNA-seq analysis of the WRKY genes by Massa [43], 22 genes were selected for analysis in our research (Fig 5). Drought, salt, heat (35°C) and SA treatments were the stress conditions.

**Fig 6:**
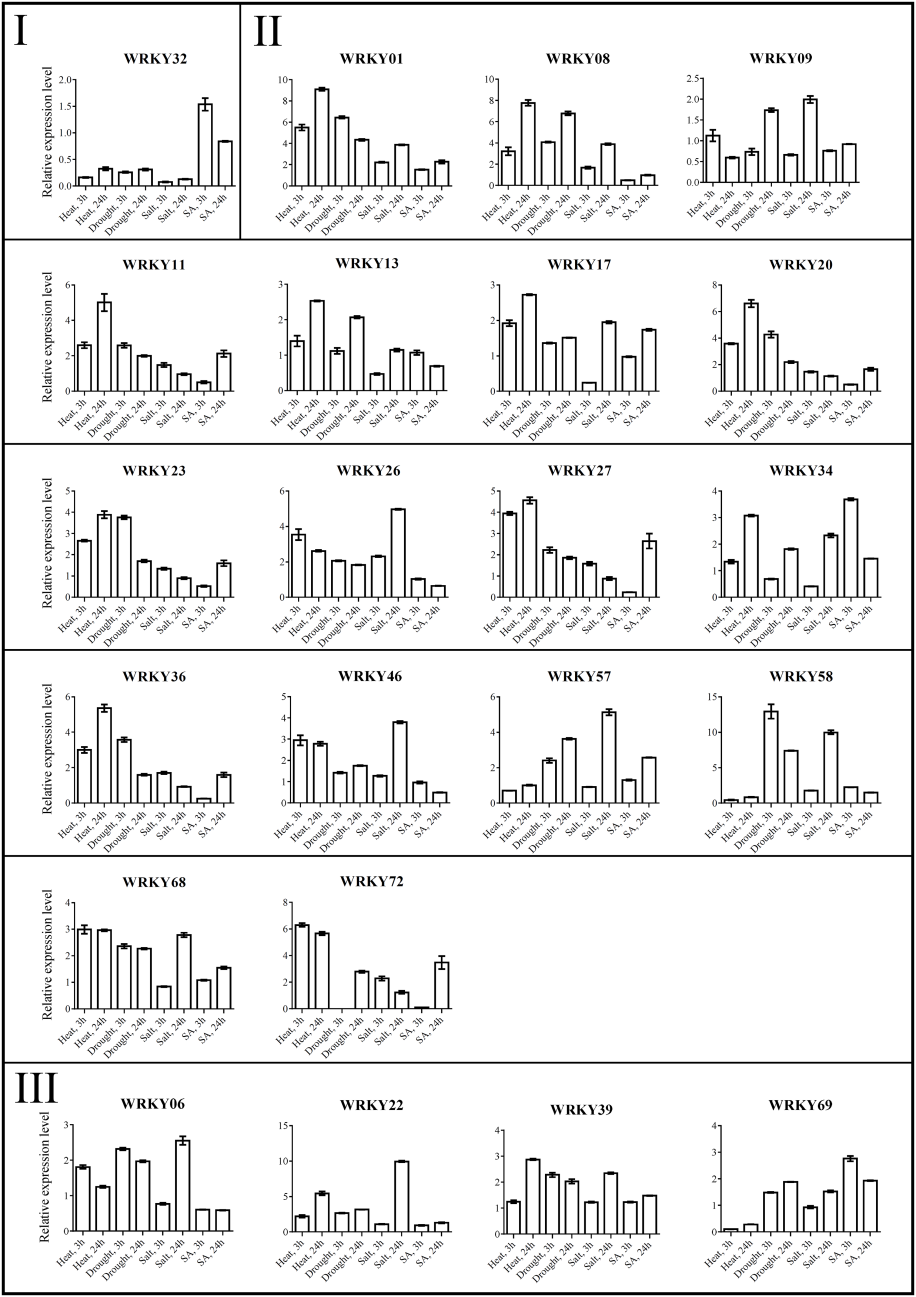
Expression analyzes 22 *StWRKY* genes under different stresses. SA means salicylic acid. The data lower than 1.0 represent the down-regulation and higher 1.0 is up-regulation. The *StWRKY72* cannot show detectable expression under drought-treatment in 3 h, so the database is missing.

Analysis of the 22 genes under the 4 stresses showed (Fig 6) that only 2 genes (*StWRKY01* and *StWRKY39*) were up-regulation under each stress. The overwhelming majority of genes under SA stress had low expression only *StWRKY34* under 3 h treatment and *StWRKY72* under 24 h treatment had highest expression in these genes, and they all had three-fold expression compared with control. Under abiotic stresses (heat, drought and salt), almost all genes were up-regulated in 3 h or 24 h treatment except*StWRKY32*. Under the different stress treatments, *StWRKY58* had highest expression under drought and salt stresses, with twelve-fold and nearly ten-fold than control, respectively. *StWRKY01* and *StWRKY22* also had nearly ten-fold and nine-fold higher than control under the heat and salt treatment, respectively. Moreover, individual WRKY gene could respond more than one stress factor, simultaneously. That is to say, one gene could have diverse function under different stresses. *StWRKY01*, *08*, *22* and *26* responded to the abiotic stresses. *StWRKY20*, *27*, *36* and *72* were induced by drought and heat stresses. Similar conditions have also occurred in other plants *AtWRKY25* and *AtWRKY33* responded concurrently under heat and salt treatments [44] and *GmWRKY53* was induced by cold, salt and drought treatments [45].

It is common knowledge that potato originated in low-temperature environments in the Andes Mountains, and so it is suited to cold locations. Conversely, warm environments could be a negative factor in plant development.

Thus, heat treatment was used to search for regulating genes in the WRKY family. For example, overexpressing *OsWRKY11* under the control of HSP101 promoter enhanced heat tolerance [46]. In our study, only 4 of 22 were down-regulated following 3 h and 24 h under heat treatment. Seventeen of 22 genes enhanced their expression. Of these, almost all of genes showed higher expression after 24 h and only *StWRKY09* turned down-regulated by 24 h. *StWRKY32*, *58* and *69* were down-regulated at both times. *StWRKY01*, *08*, *20* and *22* were significantly up-regulation after 24 h treatment compared with 3 h.

Drought is usually related to salinity and they have a combined impact on plant growth, development and productivity. Thus, understanding drought and salinity regulations is important for yield. The WRKY family is well known to respond to a variety of stresses [47]. Almost all genes were up-regulated in 24 h of drought treatment except for *StWRKY32*. Only *StWRKY09*, *32* and *34* were down-regulated at 3 h. *StWRKY72* could not show detectable expression. Significantly enhanced expression occurred for *StWRKY58*.

Following salt treatment, thirteen of 22 genes were up-regulated after 3 h of treatment and seventeen of 22 genes were up-regulated after 24 h. The highest expression genes were *StWRKY22* and *58* in 24 h treatments. However, up-regulation of the WRKY family genes in potato showed little increase after 3 h treatment. After 24 h treatment, the expression increased and only five of 22 genes (*StWRKY11*, *23*, *27*, *32* and 36) were lower than controls. Interestingly, *StWRKY32* always maintained down-regulation and showed less than one-tenth of control expression. In these 22 genes, the expression of *StWRKY22* and *StWRKY58* were greatly enhanced till 24 h treatment.

SA can be a key signaling molecule in response to biotic stresses. Infection of plants causes rapid increase of SA level that leads to the expression of genes encoding the pathogen proteins and the activating disease resistance [48]. That was why SA was chosen as a stress treatment in this search. The genes expression showed that nearly all genes were the same as control, and some genes were even down-regulated. Only seven of 22 genes were up-regulated. The highest expression was just below four-fold compared with control. Interestingly, *StWRKY72* was highly up-regulated under 24 h treatment, but had lower expression at 3 h, its homologous genes were down-regulated at both times [32, 49].

### *Cis*-elements in the promoter regions of *StWRKY* genes

Regulatory elements are essential to control gene expression [50]. To further understand the response of *StWRKY* genes under the various stress treatments, the *cis*-elements in 22 *StWRKY* genes which were used for qRT-PCR analysis was predicted their elements which were related with various stress in promoter regions. The 1500-bp upstream promoter regions were searched in PlantCARE, and the elements were showed in S1 Fig, including ABRE element (ABA responsiveness) found in eight promoter regions of selected *StWRKY* genes. Interestingly, *StWRKY*57 contained six ABRE elements and this gene can respond to the abscisic acid (ABA) signaling pathway. MBS element (drought-inducibility) occurred in the promoter region of 12 genes. Previous research has shown two signaling pathways in response to drought stress: ABA-dependent and ABA-independent [51]. Thus, two elements, ABRE and MBS could respond to drought stress. The HSE element (heat stress responsiveness) existed in the promoter area of 17 *StWRKY*s, and of these *StWRKY*58 had more HSEs (about 7) and may be related to heat stress resistance. TC-rich repeats element (*cis*-acting element involved in defense and stress responsiveness) was the most element in the promoter region of 22 *StWRKY*s, with about 18 genes containing this element. Fourteen promoters had a TCA-element (SA responsiveness) and only 10 promoters showed a W-box element (WRKY binding site).

## Discussion

The WRKY TFs are among the most important regulatory network members in plants and play pivotal roles in abiotic [52] and biotic stress responses. [53]. Considerable research has been performed on the functions of WRKY TFs, and some WRKY genes have been cloned to analyze their function [54, 55]. However, there is little relevant information from the world’s fourth most-important staple crop, potato. [29].

A total of 79 genes were found to encode the WRKY family. The majority of sequences were located on chromosomes, and only one of the *StWRKY* genes, *StWRKY79* was not located in any chromosome according to the data from the internet. That would proof that the potato whole-genome sequencing was not complement. In previous research, several genes were also not located in any chromosomes [13, 56].

Research in *A*. *thaliana* has shown that duplication might affect gene family size and distribution. Tandem duplication influences expansion and segmental duplication influences evolution and functional prediction [57]. The *StWRKY* genes showed 15 duplications: eight tandem and seven segmental, the *CaWRKY* genes showed 11 tandem clusters [35], *AdWRKY* genes had four tandem clusters and seven segmental duplications, and *AiWRKY* genes had six tandem clusters and 10 segmental duplications [58]. Thus, a number of other higher plants showed WRKYs with duplications.

Usually, WRKY genes can be divided into three main groups, and later research in higher plants showed that WRKY Group II consisted of five sub-groups [10]. WRKY genes may also have four major lineages comprising sub-groups II-a and II-b, sub-groups II-d and II-e, sub-group II-c and Group I, and Group III alone [56]. This conclusion was confirmed by Rinerson’s research [36]. Wang [42] reported that Group III genes could influence disease resistance [59] and promoted biology evolution and good adaptability [10]. We found that 14 WRKY genes in potato belonged to Group III.

The other mainly evidence concerning WRKY family classification was the variety of motifs. Almost all WRKY genes contained the conserved WRKYGQK domain. The zinc finger structures are an important part of the WRKY TFs, as clearly shown by motifs research (Fig 4C and 4D). Each group was constituted of different motifs. Group I had two WRKY domains, motifs 1 and 6. Almost every gene in Group I contained by motifs 6, 13 and 7 from N-terminal and motifs 5, 1 (6), 3 (13), 4 and 2 from C-terminal. Others contained motifs 1‒4, which was the main structure of the WRKY family. Motifs 11 and 14 together appeared in Group III. Sub-groups II-a and II-b contained motifs 8 and 11, sub-groups II-d and II-e contained motif 15, and sub-group II-c contained motif 5 these could show the characteristics of each group. This distribution further illustrated the relationships with each group.

Comparing DNA sequences and transcription sequences, DNA sequences possessed more amino acids. So gene models consisted of two parts of genome sequences [60]: the exon, which results in protein, and the intron, which is lost in transcription sequences. Although introns are not translated they contain many elements to regulate gene expression [61]. The introns were located between codons named phase 0, and after the first or second nucleotides of codons named phase 1 or 2. These two kinds of introns were found in nine *StWRKY* genes. Interestingly, eight genes belonged to Group I. Analysis of tandem duplication genes showed that they also had same intron-exon structures.

The WRKY TF family occurs widely and has been researched in many plants. Flowering plants have a larger WRKY family, and these TFs have important functions [37]. Various stresses affect the yield and the WRKY family responds to many of these stresses [62].

Many studies have investigated salt, heat and other abiotic stresses and the response of WRKY genes [54, 62]. Biotic stresses [63] are also of wide concern and salicylic acid, abscisic acid, methyl jasmonate treatments can be used to represent biotic stresses [55]. In this study, salt-, heat-, drought- and SA-response mechanisms were the main research objects.

Under the three abiotic stresses, heat, salt and drought, *StWRKY32* was all down-regulated, but in *Brachypodium distachyon* [32], *Fragaria vesca* [49] and *C*. *annuum* [35], its homologs *BdWRKY61*, *FvWRKY24* and *CaWRKY3* were up-regulated under heat and drought, and unchanged under salt stress, compared with controls. *StWRKY58* was up-regulated with salt and drought treatments and down-regulated with heat, its homologs *BdWRKY14*, *FvWRKY46* and *CaWRKY10* were all down-regulated. In different plants the WRKY genes might have different functions. Interestingly, *StWRKY22* expression was most enhanced after 24 h of salt treatment, and its homologs in soybean *GmWRKY96* and in *A*. *thaliana AtWRKY40* were also reported to show enhanced expression under salt treatment [1, 4]. *StWRKY01* was up-regulated similarly to the homolog gene *BdWRKY16*. Thus, *StWRKY22* and *StWRKY58* had high expression revealing their importance in response to salt stress, *StWRKY01* was relevant to heat resistance and *StWRKY58* also had hightest expression under drought treatment.

More genes responded to drought stress compared with the other treatments. The initial analyses suggest that *StWRKY* genes may form a core component of drought stress response. *StWRKY01* and *StWRKY58* are candidate genes for further functional research and their roles in heat or drought stress signaling should be verified.

## Conclusions

We found a total of 79 genes of the WRKY TF family located in 11 of 12 chromosomes of potato. Phylogenetic analysis divided these genes into three groups, with five sub-groups comprising Group II. The potato virus-free seedlings were treated with heat, salinity, drought and SA, and *StWRKY* gene expression monitored. qRT-PCR of 22 *StWRKY* genes in response to stresses, and database analysis, confirmed strong responses of WRKY genes, especially to drought and heat. Thus, we identified some interesting candidate WRKY genes for future functional analysis.

## Supporting information

S1 Table The design of primer sequences used for RT-PCR. (DOC)

S2 Table The data of expression level in *StWRKY* genes. (DOC)

S1 Fig *Cis*-elements distribute the 22 *StWRKY* genes’ promoter regions. The cycle from outside-to-inside indicated different elements, ABRE, HSE, MBS, TC-rich repeats, TCA-element and W-box. The different color represented the various genes. And the different numbers in each boxes meant the quantity of *cis*-elements.
(TIF)
