# Supplementary material for: Genome-wide identification of the potato WRKY transcription factor family
Source: PLoS One. 2017 Jul 20;12(7):e0181573. doi: 10.1371/journal.pone.0181573 (PMC5519183; doi:10.1371/journal.pone.0181573)
Supplement: S1 Table — (DOC) [file pone.0181573.s001.doc]

Table S1. Primer sequences used in RT-PCR

| Gene | Forward (5' to 3') | Reverse (5' to 3') |
| --- | --- | --- |
| WRKY01 | AGACACCAACATTACCACCTA | TTGATGGCGATAACATTCC |
| WRKY06 | CAAGGGCTTATTATAGGTG | CAACTGTGCCTTCCTTTAT |
| WRKY09 | CGCAAACAGGTTCAAAGGT | GCTGAAATAGTCGCCATAC |
| WRKY11 | GCTCAGTGGCTCCATTAT | GAACCCGAAAGAAGCATA |
| WRKY13 | GTTCCGGTTTGTTCAATG | CAAGAATCAGATGGTGGC |
| WRKY17 | AAGTACAGCGCCTTTCCA | GGTATGGGAATGGGAGGA |
| WRKY22 | TGCCCGTAGCAATACATC | ATGGCACATTGTTAGGAT |
| WRKY23 | ACTTATAGAGGCCAACATA | AGTAGAGGTAGAAGAGGGA |
| WRKY26 | ACTCTTGATTTGGCTGAAC | TCCTGGTGATTTACGCTTG |
| WRKY27 | CCACGAACCGTAGTAATCT | ACAACTCTTGAACGCCTAA |
| WRKY32 | GGCATCCCAGGTTCAGTCT | GGTTTGTCAACAGCGAAGG |
| WRKY34 | AGCATCATCGTCGTCATC | CATTAGTCCCAAGAACCC |
| WRKY36 | AAAAGCCCAAAGGATGAG | CCCGATTTCAAGTGGAGG |
| WRKY39 | GCGAAACAGAGGAGTCATT | CTGGTAAAGGCGAAGAAGC |
| WRKY46 | TGATAGAGCAAATGGCTAC | TTTACCATCTCCCTGTCTG |
| WRKY57 | TCCCTCCGATCAAACCTC | TTGACTGGGCATGTTGGT |
| WRKY58 | GCCCAGTCAAGAAGAAGG | GTTAAGGAAGAAGCCATTT |
| WRKY68 | ATACCCAAGCAGGGACGAA | TAGCCAAGGGAACTGAAA |
| WRKY69 | AAAGAGGACGATACAAGA | CCTAAATAGTGGTGGATT |
| WRKY72 | TCTCCAATAATCCCAACT | CACCTCCTCTTTACCATC |

Primers were designed by Primer premier 5
