# Supplementary material for: Genome-wide identification of the potato WRKY transcription factor family
Source: PLoS One. 2017 Jul 20;12(7):e0181573. doi: 10.1371/journal.pone.0181573 (PMC5519183; doi:10.1371/journal.pone.0181573)
Supplement: S2 Table — (DOC) [file pone.0181573.s002.doc]

Table S2. The expression database of *StWRKY* genes

| Gene | Salt,3h | Salt,24h | Heat,3h | Heat,24h | Drought,3h | Drought,24h | SA,3h | SA,24h |
| --- | --- | --- | --- | --- | --- | --- | --- | --- |
| StWRKY01 | 2.222566 | 3.868476 | 5.497864 | 9.097013 | 6.440982 | 4.336806 | 1.532829 | 2.26749 |
| StWRKY06 | 0.771878 | 2.554637 | 1.809736 | 1.25236 | 2.316351 | 1.970709 | 0.605697 | 0.588714 |
| StWRKY08 | 1.682909 | 3.882343 | 3.218333 | 7.770672 | 4.083048 | 6.77905 | 0.501809 | 0.971337 |
| StWRKY09 | 0.659634 | 1.992315 | 1.121964 | 0.594407 | 0.735156 | 1.734776 | 0.76018 | 0.917626 |
| StWRKY11 | 1.480971 | 0.960486 | 2.595845 | 5.006791 | 2.584415 | 1.985909 | 0.505337 | 2.122157 |
| StWRKY13 | 0.46905 | 1.143835 | 1.397603 | 2.531764 | 1.118905 | 2.069858 | 1.072611 | 0.691044 |
| StWRKY17 | 0.242755 | 1.948835 | 1.921142 | 2.726053 | 1.362598 | 1.512809 | 0.977468 | 1.736752 |
| StWRKY20 | 1.455469 | 1.134223 | 3.577212 | 6.602555 | 4.268319 | 2.199842 | 0.50076 | 1.651094 |
| StWRKY22 | 1.097646 | 9.942111 | 2.209979 | 5.441952 | 2.677072 | 3.165099 | 0.941061 | 1.302656 |
| StWRKY23 | 1.33976 | 0.894699 | 2.660352 | 3.883118 | 3.761208 | 1.700006 | 0.518529 | 1.599545 |
| StWRKY26 | 2.311118 | 4.973386 | 3.543068 | 2.618023 | 2.060231 | 1.832964 | 1.031894 | 0.643148 |
| StWRKY27 | 1.58115 | 0.883154 | 3.943135 | 4.560846 | 2.219735 | 1.854175 | 0.242579 | 2.644486 |
| StWRKY32 | 0.077388 | 0.129127 | 0.161889 | 0.326562 | 0.261261 | 0.311508 | 1.536157 | 0.839614 |
| StWRKY34 | 0.415704 | 2.327192 | 1.338441 | 3.075984 | 0.687825 | 1.816212 | 3.685439 | 1.455629 |
| StWRKY36 | 1.699041 | 0.924214 | 2.997416 | 5.357314 | 3.570845 | 1.594394 | 0.254349 | 1.594638 |
| StWRKY39 | 1.229699 | 2.345075 | 1.244495 | 2.876524 | 2.280418 | 2.034383 | 1.23219 | 1.482424 |
| StWRKY46 | 1.266724 | 3.7994 | 2.948592 | 2.780866 | 1.420147 | 1.751425 | 0.963325 | 0.493464 |
| StWRKY57 | 0.91547 | 5.132319 | 0.708606 | 1.009338 | 2.409024 | 3.629485 | 1.306467 | 2.571726 |
| StWRKY58 | 1.787541 | 9.986446 | 0.442465 | 0.841357 | 12.92953 | 7.406522 | 2.241289 | 1.506879 |
| StWRKY68 | 0.841261 | 2.781696 | 2.988644 | 2.957502 | 2.359382 | 2.26361 | 1.080406 | 1.547858 |
| StWRKY69 | 0.931109 | 1.524007 | 0.107822 | 0.281288 | 1.482944 | 1.885396 | 2.76088 | 1.929809 |
| StWRKY72 | 2.286188 | 1.239562 | 6.301853 | 5.66038 |  | 2.786725 | 0.099949 | 3.474352 |
