# Supplementary figures and images for: Genome-wide identification of the potato WRKY transcription factor family
Source: PLoS One. 2017 Jul 20;12(7):e0181573. doi: 10.1371/journal.pone.0181573 (PMC5519183; doi:10.1371/journal.pone.0181573)

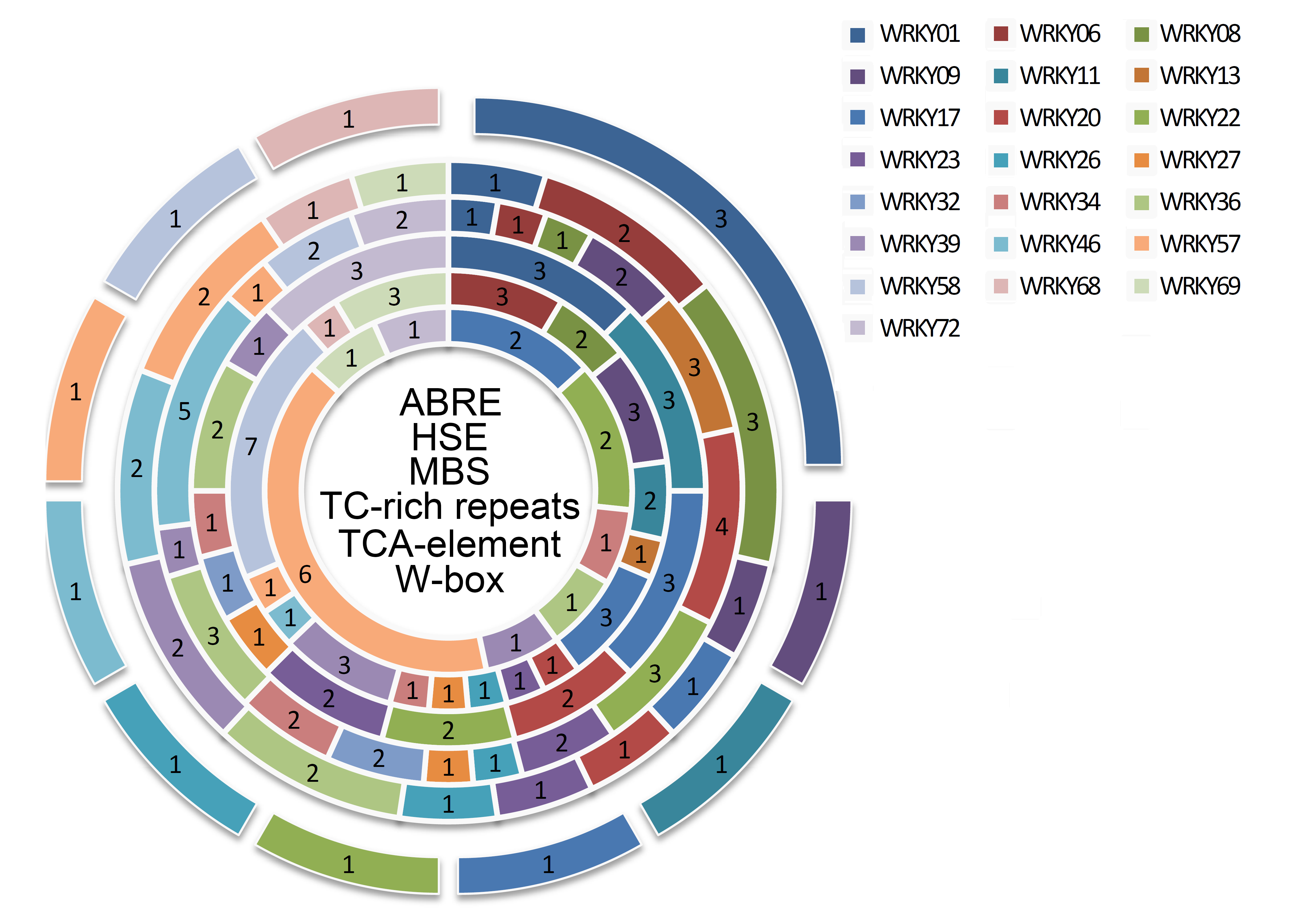

Supplement: S1 Fig — The cycle from outside-to-inside indicated different elements, ABRE, HSE, MBS, TC-rich repeats, TCA-element and W-box. The different color represented the various genes. And the different numbers in each boxes meant the quantity of cis-elements. (TIF) [file pone.0181573.s003.TIF]
